# Preclinical Evaluation of the Novel Small-Molecule MSI-N1014 for Treating Drug-Resistant Colon Cancer via the LGR5/β-catenin/miR-142-3p Network and Reducing Cancer-Associated Fibroblast Transformation

**DOI:** 10.3390/cancers12061590

**Published:** 2020-06-16

**Authors:** Vijesh Kumar Yadav, Yan-Jiun Huang, Thomashire Anita George, Po-Li Wei, Maryam rachmawati Sumitra, Ching-Liang Ho, Tzu-Hao Chang, Alexander T. H. Wu, Hsu-Shan Huang

**Affiliations:** 1 The Program for Translational Medicine, Graduate Institute of Biomedical Informatics, College of Medical Science and Technology, Taipei Medical University, Taipei 11031, Taiwan; vijeshp2@gmail.com; 2 Graduate Institute of Biomedical Informatics, College of Medical Science and Technology, Taipei Medical University, Taipei 11031, Taiwan; kevinchang@tmu.edu.tw; 3 Division of Colorectal Surgery, Department of Surgery, Taipei Medical University Hospital, Taipei Medical University, Taipei 11031, Taiwan; colorectalman@gmail.com (Y.-J.H.); poliwei@tmu.edu.tw (P.-L.W.); 4 Department of Surgery, College of Medicine, Taipei Medical University, Taipei 11031, Taiwan; 5 International PhD Program in Biomedical Engineering, College of Biomedical Engineering, Taipei Medical University, Taipei 11031, Taiwan; thomashireg@gmail.com; 6 Graduate Institute for Cancer Biology & Drug Discovery, College of Medical Science and Technology, Taipei Medical University, Taipei 11031, Taiwan; maryamrachma60@gmail.com; 7 Division of Hematology and Oncology Medicine, Department of Internal Medicine, Tri-Service General Hospital, National Defense Medical Center, Taipei 114, Taiwan; d204097002liu@gmail.com; 8 Clinical Big Data Research Center, Taipei Medical University Hospital, Taipei 11031, Taiwan; 9 Graduate Institute of Medical Sciences, National Defense Medical Center, Taipei 114, Taiwan; 10 The Ph.D. Program for Translational Medicine, College of Medical Science and Technology, Taipei Medical University and Academia Sinica, Taipei 11031, Taiwan; 11 Ph.D. Program in Biotechnology Research and Development, College of Pharmacy, Taipei Medical University, Taipei 11031, Taiwan; 12 School of Pharmacy, National Defense Medical Center, Taipei 114, Taiwan

**Keywords:** colon cancer, drug resistance, small-molecule therapeutics, cancer-associated fibroblasts (CAFs), cancer stemness, miR-142

## Abstract

Colorectal cancer represents one of the most prevalent malignancies globally, with an estimated 140,000 new cases in the United States alone in 2019. Despite advancements in interventions, drug resistance occurs in virtually all patients diagnosed with late stages of colon cancer. Amplified epidermal growth factor receptor (EGFR) signaling is one of the most prevalent oncogenic drivers in patients and induces increased Janus kinase (JAK)/signal transduction and activator of transcription (STAT) and β-catenin functions, all of which facilitate disease progression. Equally important, cancer-associated fibroblasts (CAFs) transformed by cancer cells within the tumor microenvironment (TME) further facilitate malignancy by secreting interleukin (IL)-6 and augmenting STAT3 signaling in colon cancer cells and promoting the generation of cancer stem-like cells (CSCs). Based on these premises, single-targeted therapeutics have proven ineffective for treating malignant colon cancer, and alternative multiple-targeting agents should be explored. Herein, we synthesized a tetracyclic heterocyclic azathioxanthone, MSI-N1014, and demonstrated its therapeutic potential both in vitro and in vivo. First, we used a co-culture system to demonstrate that colon cancer cells co-cultured with CAFs resulted in heightened 5-fluorouracil (5-FU) resistance and tumor sphere-forming ability and increased side populations, accompanied by elevated expression of cluster of differentiation 44 (CD44), β-catenin, leucine-rich repeat-containing G-protein-coupled receptor 5 (LGR5), and ATP-binding cassette super-family G member 2 (ABCG2). MSI-N1014 suppressed cell viability, colony formation, and migration in both DLD1 and HCT116 cells. MSI-N1014 treatment led to decreased expressions of oncogenic markers, including mammalian target of rapamycin (mTOR), EGFR, and IL-6 and stemness markers such as CD44, β-catenin, and LGR5. More importantly, MSI-N1014 treatment suppressed the transformation of CAFs, and was associated with decreased secretion of IL-6 and vascular endothelial growth factor (VEGF) by CAFs. Furthermore, MSI-N1014 treatment resulted in significantly reduced oncogenic properties, namely the migratory ability, tumor-sphere generation, and resistance against 5-FU. Notably, an increased level of the tumor suppressor, miR-142-3p, whose targets include LGR5, IL-6, and ABCG2, was detected in association with MSI-N1014 treatment. Finally, we demonstrated the therapeutic potential of MSI-N1014 in vivo, where combined treatment with MSI-N1014 and 5-FU led to the lowest tumor growth, followed by MSI-N1014 only, 5-FU, and the vehicle control. Tumor samples from the MSI-N1014 group showed markedly reduced expressions of LGR5, β-catenin, IL-6, and mTOR, but increased expression of the tumor suppressor, miR-142-3p, according to qRT-PCR analysis. Collectively, we present preclinical support for the application of MSI-N1014 in treating 5-FU-resistant colon cancer cells. Further investigation is warranted to translate these findings into clinical settings.

## 1. Introduction

Colorectal cancer (CRC) ranks as one of the most prevalent gastrointestinal cancer types globally, accounting for an estimated nine percent of all cancer cases [1]. Patients with advanced stages of CRC often manifest unfavorable phenotypes, including treatment resistance and distant metastasis, which lead to limited therapeutic options. Studies have shown that the tumor microenvironment (TME) plays a key role in the development and progression of CRC. One of the major components of the CRC TME is stromal fibroblasts. A subpopulation of these fibroblasts is referred to as cancer-associated fibroblasts (CAFs), which are closely linked to the malignant characteristics of late-stage CRC [2]. CAFs are characterized by the expression of alpha-smooth muscle actin (α-SMA), which promotes the metastatic potential and stemness within the TME [3]. CAFs contribute to the progression of CRC by secreting oncogenic cytokines such as interleukin (IL)-6, transforming growth factor (TGF)-β1, and epidermal growth factor (EGF), all of which are documented to promote the epithelial-to-mesenchymal transition (EMT) and induce stemness [4,5,6,7]. Thus, preventing and inhibiting CAF transformation could provide significant therapeutic benefits.

Both experimental and clinical data indicate that amplified EGF receptor (EGFR) and Kirsten rat sarcoma (KRAS) mutations were found in approximately 50% of patients [8], and both oncogenic molecules further amplify downstream signaling such as signal transducer and activator of transcription 3 (STAT3) and mammalian target of rapamycin (mTOR), resulting in enhanced proliferation, metastasis, and stemness. Thus, the combination of bevacizumab (an anti-EGFR antibody) and chemotherapy has been trialed with the hope of improving overall survival (OS) of patients with advanced-stage CRC [9]. Modified FOLFOX6 regimen that is a combination of l-leucovorin (l-LV), 5-fluorouracil (5-FU), and Oxaliplatin (L-OHP) was approved for the first-line treatment of metastatic colorectal cancer [10]. However, recent clinical evidence revealed that adding the adjuvant bevacizumab to the FOLFOX6 chemo-regimen did not significantly improve the OS of patients with stage II/III rectal cancer [11]. This finding suggests that additional players and/or signaling networks exist to promote the survival of CRC cells. Connected to this point, current therapeutic regimens specifically target CRC cells but fail to suppress the generation of CAFs.

Previously, our group synthesized a series of tetracyclic heterocyclic azathioxanthones and showed the potential cytotoxic potential of some of those candidates [12]. Herein, we evaluate one of the candidates, named MSI-N1014, for its efficacy in suppressing CRC carcinogenesis, as well as the potential for preventing the generation of CAFs. We first demonstrated that CRC cells co-cultured with CAFs, showed increased 5-fluorouracil (5-FU) resistance, colony formation, and self-renewal ability. This was accompanied by increased expressions of oncogenic markers such as mTOR, IL-6, ATP-binding cassette super-family G member 2 (ABCG2), and TGF-β1 and stemness markers, leucine-rich repeat-containing G-protein coupled receptor 5 (LGR5), and β-catenin. In return, CAF-educated CRC cells promoted CAF transformation from normal fibroblasts. Analyses of public databases demonstrated that higher expressions of EGFR, LGR5, and IL-6 in CRC patients were associated with a significantly lower survival ratio and coincided with lower expression of miR-142-3p, a suppressor of these oncogenic markers. Essentially, CRC tumor-spheres treated with MSI-N1014 showed significantly reduced expressions of LGR5, β-catenin, and ABCG2, and reduced resistance against 5-FU, with an increased level of microRNA (miR)-142-3p, which targets both LGR5 and ABCG2. In addition, treatment with MSI-N1014 resulted in a decreased CAF-transforming ability in both DLD1 and HCT116 cells, coincident with decreased secretion of IL-6 and VEGF by CRC cells and reduced expression of α-SMA by CAFs. CAFs treated with MSI-N1014 showed reduced abilities to promote the tumor-sphere formation, the EMT, and resistance against 5-FU via increased miR-142-3p expression. Finally, we evaluated MSI-N1014’s efficacy using a mouse xenograft model and confirmed our in vitro observations. MSI-N1014 appeared to re-sensitize 5-FU-resistant CAF-educated DLD1 cells and led to the lowest tumor burden followed by the MSI-N1014 group, while the 5-FU and vehicle groups showed no significant difference.

In summary, we provided evidence that MSI-N1014 suppressed the major colon cancer stemness markers, LGR5 and β-catenin, and oncogenic signaling, such as mTOR and IL-6; it also prevented cancer cell-mediated CAF transformation. Thus, MSI-N1014 should be further investigated for its potential as a single or an adjuvant anticancer therapeutic agent for treating patients with chemo-resistant CRC.

## 2. Results

### 2.1. CAFs Increased Oncogenic Properties of CRC Cells with Increased Association of EGFR

**Figure 1:**
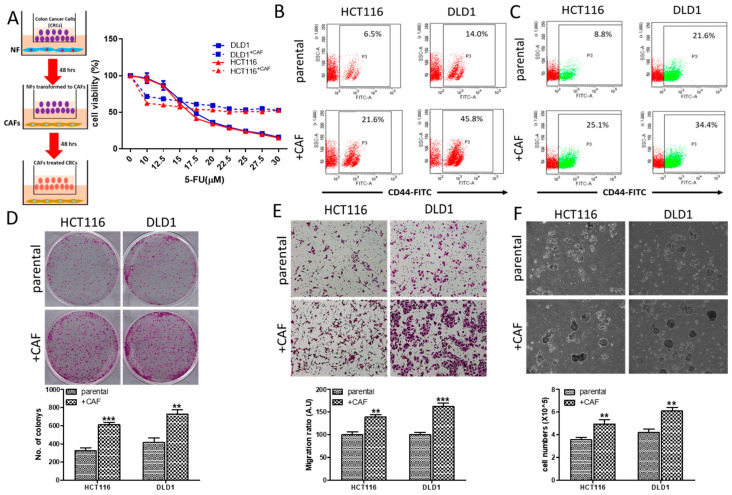
Cancer-associated fibroblasts (CAFs) increased the oncogenic properties of colon cancer cells. (**A**) Insert illustrates the co-culture system of DLD1 and HCT116 colorectal cancer (CRC) cells with Normal fibroblasts (NF) and CAFs. Cell viability assay showed increased 5-fluorouracil (5-FU) resistance in CAF-educated DLD1 and HCT116 cells, compared to their naïve counterparts. Flow cytometric analysis of DLD1 and HCT116 cells co-cultured with CAFs. Increased CD44^+^ cell population (**B**) and side population (**C**) in both DLD1 and HCT116 cells, 48 h post CAF culture. Enhanced colony-forming (**D**), migratory (**E**), and tumor sphere-generating (**F**) abilities in both CRC cell lines post CAF co-culture. ** *p* < 0.01, *** *p* < 0.001.

CAFs were implicated in the development and progression of CRC. Herein, we demonstrated the tumor-promoting roles of CAFs, where we co-cultured DLD1 and HCT116 CRC cells with CAFs (Insert, Figure 1A). First, we demonstrated that CAF-educated DLD1 and HCT116 cells are more resistant against 5-FU (Figure 1A). For instance, under the influence of CAFs, the IC_50_ value of 5-FU of DLD1 cells was approximately 2-fold higher than its naïve counterpart. Second, our flow cytometric analysis showed that CAF educated DLD1 and HCT116 cells showed increased percentages of the cluster of differentiation 44-positive (CD44^+^) cell population (Figure 1B), and more importantly, side-population (SP) cells (Figure 1C). These observations were accompanied by increased colony-forming (Figure 1D), migratory (Figure 1E), and self-renewal abilities (Figure 1F), compared to their naïve counterparts.

### 2.2. MSI-N1014 Treatment Suppressed CRC Tumorigenesis

**Figure 2:**
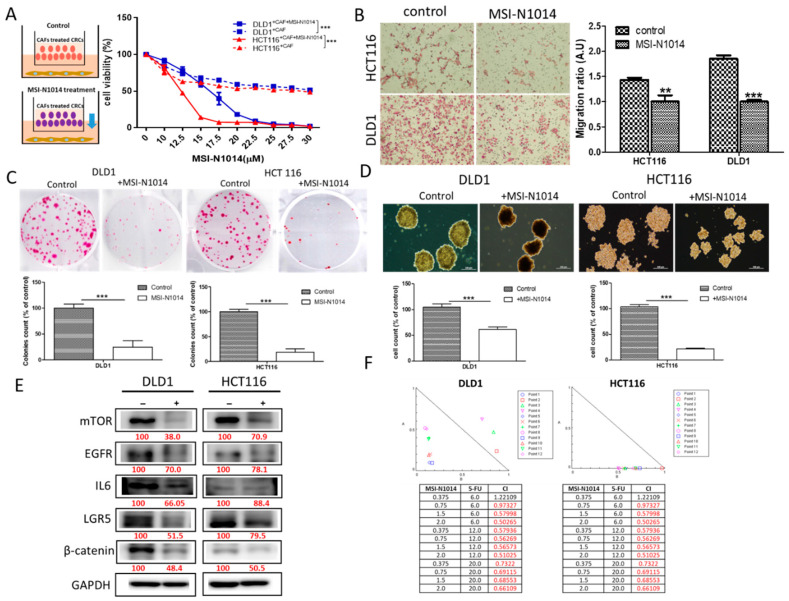
MSI-N1014 exerted anti-colorectal cancer (CRC) properties. (**A**) The insert depicts the experimental design where MSI-N1014 effect on cancer-associated fibroblast (CAF) CRC-cells were analyzed. MSI-N1014 dose-dependently reduced the cell viability of CAF-educated DLD1 and HCT116 cells. Reduced migratory (**B**), colony-forming (**C**), and tumor sphere-formation abilities (**D**) in both DLD1 and HCT116 cells post MSI-N1014 treatment. (**E**) Western blot analysis revealed reduced levels of mammalian target of rapamycin (mTOR), epidermal growth factor receptor (EGFR), interleukin (IL)-6, leucine-rich repeat-containing G-protein coupled receptor 5 (LGR5), and β-catenin in MSI-1014-treated cells compared to their control counterparts. (**F**) Isobologram analysis showing the synergistic effects of MSN-1014 and 5-fluorouracil (5-FU) were achieved in different concentration combinations in both DLD1 and HCT116 cells. Numbers in red indicate the relative expression ratio. ** *p* < 0.01, *** *p* < 0.001.

Next, we evaluated the potential therapeutic effects of MSI-N1014 in vitro. First, we demonstrated that the addition of MSI-N1014 (15 µM, 48 h) overcame 5-FU resistance in CAF-educated DLD1 and HCT116 cells (insert, Figure 2A). The presence of MSI-N1014 significantly reduced the migratory (Figure 2B), colony-forming (Figure 2C), and tumor-sphere forming abilities (Figure 2D). These phenomena were accompanied by marked reductions in expressions of oncogenic/stemness markers such as LGR5, β-catenin, EGFR, and mTOR, as well as the IL-6 inflammatory marker (Figure 2E). More importantly, we found that MSI-N1014 and 5-FU synergistically (CI < 1) reduced the viability of DLD1 and HCT116 cells (Figure 2F).

### 2.3. MSI-N1014 Treatments Lowered CRC’s Ability to Generate CAFs

**Figure 3:**
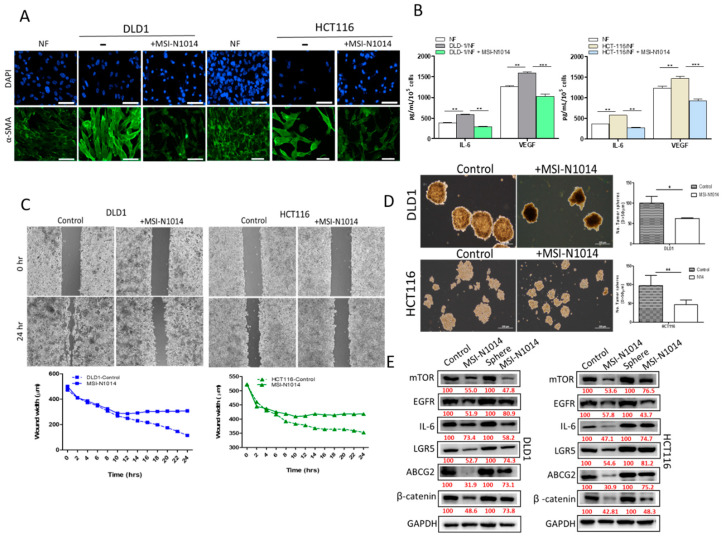
MSI-N1014 treatment prevented cancer-associated fibroblast (CAF) transformation. (**A**) Representative immunofluorescence images of CAFs transformed by MSI-1014-treated DLD1 and HCT116 cells. Reduced expression of alpha-smooth muscle actin (α-SMA) was observed. (**B**) ELISAs showed reductions in interleukin (IL)-6, and vascular endothelial growth factor (VEGF) released by CAFs generated by co-culturing with MSI-N1014-treated DLD1 and HCT116 cells. MSN-N1014-treated CRC cells showed reduced migratory (**C**) and tumor sphere-generating abilities (**D**). (**E**) Western blot analysis showing that there were increased expressions of epidermal growth factor receptor (EGFR), mammalian target of rapamycin (mTOR) (oncogenic markers), leucine-rich repeat-containing G-protein coupled receptor 5 (LGR5), and β-catenin (stemness markers), and increased ATP-binding cassette super-family G member 2 (ABCG2) expressions and IL-6 (an inflammatory marker) in CRC and tumor-sphere cells. However, after MSI-N1014 treatment, these markers were significantly suppressed. Numbers in red indicate the relative expression ratio. * *p* < 0.05, ** *p* < 0.01, *** *p* < 0.001.

CAFs represent one of the major culprits within the TME that facilitates the progression of colon cancer [13]. Herein, we examined whether MSI-N1014 treatment could prevent CAF transformation. We showed that MSI-N1014 treatment of DLD1 and HCT116 cells resulted in a significantly lower ability to transform normal fibroblasts into CAFs, compared to the untreated counterparts (Figure 3A). The resultant CAFs from the MSI-N1014 group showed markedly reduced expression of α-SMA. In addition, MSI-N1014 treatment resulted in significantly reduced release of IL-6 and VEGF by CAFs (Figure 3B). More importantly, MSI-N1014-treated CRCs cells also showed a significantly lower wound-healing ability, i.e., less migration (time-lapsed video of wound healing captured shown in Supplementary Video S1) (Figure 3C) and significantly lower ability to generate tumor-spheres (Figure 3D) as compared to their control counterparts. Protein analysis by Western blotting supported these observations as there were increased expressions of the oncogenic markers, EGFR and mTOR, and stemness markers, LGR5 and β-catenin, as well as increased expressions of ABCG2 and the IL-6 inflammatory cytokine on CRC cells. However, after MSI-N1014 treatment, it reduces the expression of the markers, as mentioned earlier (Figure 3E), suggesting that MSI-N1014 effectively targets CRC cells and tumor-spheres.

### 2.4. MSI-N1014’s Anti-CRC Function Was Associated with the Induction of miR-142-3p and Reductions of Its Oncogenic Targets

**Figure 4:**
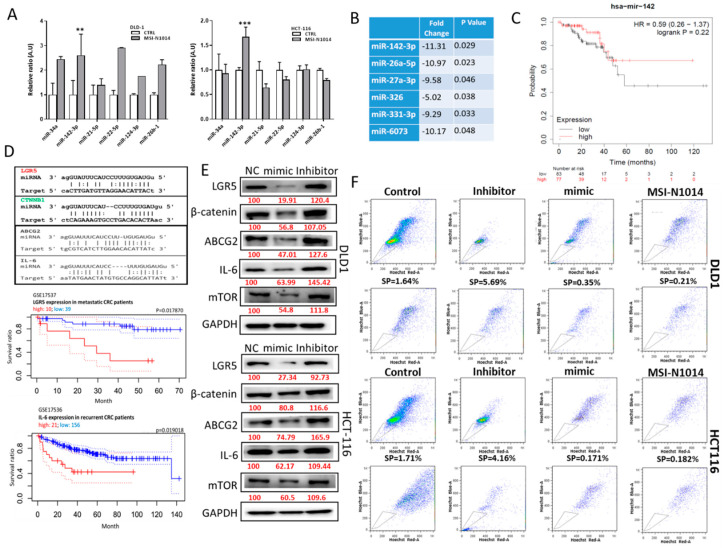
MSI-N1014 treatment induced the tumor suppressor, miR-142-3p. (**A**) Small-scale microRNA (miR) screening for MSI-N1014-treated colorectal cancer (CRC) cells. Among different miRs tested, the miR-142-3p level had significantly increased in both HCT116 and DLD1 cells after MSI-N1014 treatment. (**B**) Tabulated plasma miR profiles between CRC and normal samples [13]. A significantly reduced level of miR-142-3p in plasma collected from patients with CRC (*n* = 61), compared to normal subjects (*n* = 24) was identified. (**C**) Kaplan-Meier survival curve of a TCGA cohort suggested that a higher miR-142 was positively correlated (*p* = 0.22) with a higher survival ratio in patients with CRC. (**D**) Table listing potential targets of miR-142-3p. Leucine-rich repeat-containing G-protein coupled receptor 5 (LGR5) was predicted and experimentally validated as a strong target for miR-142-3p, while CTNNB1 (β-catenin), interleukin (IL)-6, and ATP-binding cassette super-family G member 2 (ABCG2) were also predicated targets for miR-142-3p. (**E**) Western blot analysis validating targets of miR-142-3p. Both DLD1 and HCT116 cells were transfected with miR-142-3p-mimic and inhibitor molecules. Mimic transfected samples showed markedly reduced expressions of LGR5, β-catenin, ABCG2, mammalian target of rapamycin (mTOR), and IL-6, while the opposite occurred in inhibitor-transfected samples. (**F**) Side-population (SP) flow cytometric assay. Percentages of SP cells were prominently reduced in miR-142-3p-transfected DLD1 and HCT116 cells, while they were increased in the inhibitor-transfected counterparts. Numbers in red indicate the relative expression ratio. ** *p* < 0.01, *** *p* < 0.001.

Non-coding RNA molecules have gained much traction recently in the field of oncology, especially miRs [14]. In a small panel of miRs implicated in cancer biology, we found that the miR-142-3p level was significantly induced after MSI-N1014 treatment in both DLD1 and HCT116 cells (Figure 4A). A small database analysis [15] revealed that miR-142-3p was present in significantly lower amounts in the plasma of patients with CRC (*n* = 61) compared to their normal counterparts (*n* = 24) (Figure 4B). Subsequently, The Cancer Genome Atlas (TCGA) database search indicated that elevated miR-142-3p in patients with CRC was correlated with a better survival ratio (Figure 4C). We then searched for its targets and identified several key oncogenic markers including LGR5, β-catenin, ABCG2, and IL-6, as evidenced by their binding of 3’ untranslated region (UTR) sites to miR-142-3p (Figure 4D). In addition, a lower survival ratio was strongly associated with a higher expression of LRG5 in patients with metastatic colon cancer [16] and IL-6 in recurrent colon cancer [17]. Consistently, LGR5 and IL-6 were molecular targets of miR-142-3p, as demonstrated by Western blots of HCT116 and DLD1 cells respectively transfected with miR-142-3p mimic and inhibitor molecules. Exogenous miR-142-3p mimic molecules led to decreased expressions of the predicted targets, namely, LGR5, β-catenin, IL-6, and mTOR in both DLD1 and HCT116 cells (Figure 4E); contrary observations were made when cells were transfected with a miR-142-3p inhibitor (Figure 4E). We also performed an SP assay, where the addition of miR142-3p mimic molecules led to a markedly reduced percentage of SP cells, while the reverse was true in the case of the miR-142-3p inhibitor (Figure 4F). Notably, treatment with MSI-N1014 resulted in a similar SP-suppressive effect as that of miR-142-3p mimic molecules (Figure 4F).

### 2.5. MSI-N1014 Treatment Increased 5-FU Efficacy In Vivo

**Figure 5:**
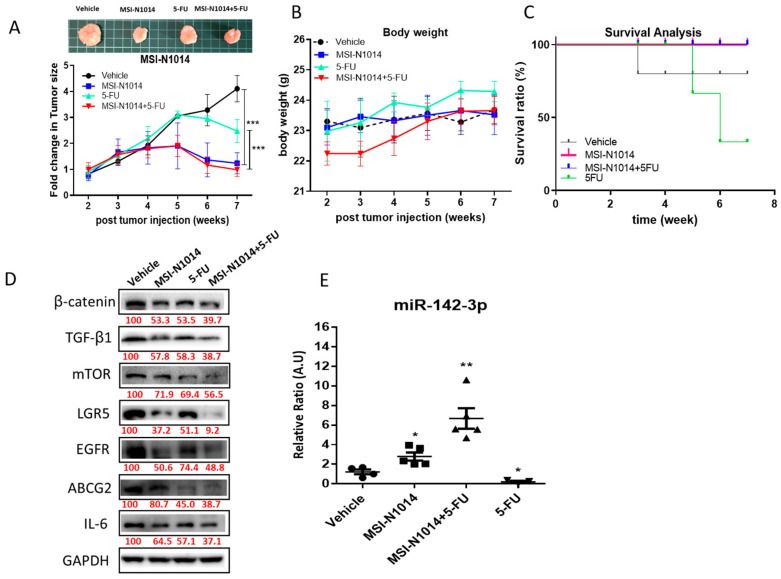
Efficacy evaluation of MSN-N1014 using a cancer-associated fibroblast (CAF)-educated DLD1 xenograft mouse model. (**A**) Tumor size over time curve. The tumor growth delay was most significant in the MSI-N1014+5-fluorouracil (5-FU) treatment group, followed by the MSI-N1014-only group, while the 5-FU-only and vehicle control groups did not show a significant difference. The insert shows representative photographs of tumor samples from each group. (**B**) Average body weight over time curve. No significant differences were observed among the different groups. (**C**) Kaplan-Meier survival curve. Mice receiving MSI-N1014 only and the combination MSI-N1014+5-FU regimen showed the highest survival ratios, while 5-FU-only and vehicle mice showed the lowest survival ratios. (**D**) Tumor sample Western blot analysis. Expressions of key molecules associated with oncogenic (epidermal growth factor receptor (EGFR) and mammalian target of rapamycin (mTOR)), stemness-associated (leucine-rich repeat-containing G-protein coupled receptor 5 (LGR5) and β-catenin), drug resistance (ATP-binding cassette super-family G member 2 (ABCG2)), and inflammatory cytokines (transforming growth factor (TGF)-β1 and interleukin (IL)-6) were clearly lower in samples from MSI-N1014+5-FU and MSI-N1014-treated tumors. (**E**) qPCR analyses of plasma levels of miR-142-3p. Pooled blood samples from all four groups of mice were analyzed for miR-142-3p plasma levels. The combination group showed the highest level followed by NSI-N1014 alone. Numbers in red indicate the relative expression ratio. * *p* < 0.05; ** *p* < 0.01.

After establishing MSI-N1014 anti-CRC functions in vitro, we then evaluated its effects using a xenograft mouse CAF-educated DLD1 tumor model. The tumor size over time clearly showed that MSI-N1014 treatment alone resulted in significantly delayed tumorigenesis, while the vehicle and 5-FU-alone groups showed no significant differences in tumor growth. Notably, MSN-1014 alone and the combination of MSN-1014 and 5-FU led to the most significant delays in tumorigenesis (Figure 5A). The body weight (BW) over time curve also indicated that no treatment regimens caused obvious/acute systemic toxicity to the animals (Figure 5B). Using a Kaplan-Meier survival curve, we verified that MSN-1014 alone or in combination with 5-FU conferred a significant survival advantage in mice, compared to the 5-FU-alone or vehicle-treated groups (Figure 5C). Comparative Western blots from tumor samples collected in all groups demonstrated reduced expressions of oncogenic markers (EGFR and mTOR), stemness markers (LGR5 and β-catenin), and the ABCG2 transporter and inflammatory cytokines (TGF-β1 and IL-6) (Figure 5D); while the qPCR analysis of plasma levels of miR-142-3p showed the highest level in MSI-N1014+5-FU-treated pooled blood samples, followed by MSI-N1014, 5-FU, and the vehicle control (Figure 5E).

## 3. Discussion

TMEs have recently garnered increased attention in the development and progression of tumor cells [18]. The presence of CAFs was identified in many cancer types, including colon cancer, and they were indicated to contribute to the development of malignant properties such as distant metastasis and drug resistance [19,20]. Thus, it is no longer sufficient to target cancer cells when it comes to developing therapeutics. Drugs that have the ability to suppress both tumor-promoting signaling and CAF generation will definitely provide superior therapeutic effects compared to traditional targeted therapeutic compounds.

The present study first provides strong evidence that CAFs promote colon tumorigenic properties when co-cultured, establishing a drug screening platform. It was clear that CAF-educated DLD1 and HCT116 cells were significantly more tumorigenic, as featured by the increased abilities to resist 5-FU, form colonies, and tumor-spheres, and migrate; these increased tumorigenic properties were associated with increased expressions of LGR5, β-catenin, and mTOR signaling. In support, previous studies indicated that increased LGR5 expression was identified in drug-resistant colon cancer cells and was a key player in generating/maintaining colon cancer stem cells [21,22]. More importantly, LGR5 was shown to potentiate Wnt/β-catenin, while TGF-β1 facilitates LGR5 expression, creating a cascading loop to initiate and amplify carcinogenesis [23,24,25,26]. Collectively, MSI-N1014 ability to suppress multiple markers, including LGR5, β-catenin, mTOR, and IL-6, could be a major factor in overcoming 5-FU resistance.

One of the major signaling routes involved in generating CAFs within the TME is TGF-β1 secreted by tumor cells [20]. As demonstrated in our normal fibroblast (NF) and colon cancer cell co-culture experiments, the addition of MSI-N1014 significantly reduced the ability of colon cancer cells to transform NFs into CAFs in part by reducing TGF-β1 and IL-6 expressions, both of which were shown to facilitate CAF transformation [27]. Equally important, CAFs transformed under MSI-N1014 treatment resulted in a lower ability to promote tumor-sphere formation and 5-FU resistance, in part by reducing their secretion of IL-6 and VEGF. It is well established that CAFs play a key role in promoting metastasis via secreting a spectrum of proangiogenic and protumorigenic cytokines, including IL-6 and VEGF [28,29,30]. Our observation that MSI-N1014 treatment led to reduced CAF transformation and secretion of IL-6 and VEGF further provides support for the potential TME-modulating and therapeutic functions of MSI-N1014.

We further examined the potential mechanistic explanations for MSI-N1014’s anti-CRC functions. Through bioinformatics and in vitro validation, we found that one of the major tumor suppressors, miR-142-3p, had significantly increased after MSI-N1014 treatment in both DLD1 and HCT116 cells. Previous evidence showed that miR-142-3p functions to suppress both tumorigenesis and the generation of cancer stem cells in triple-negative breast cancer [31,32], where overexpression of miR-142-3p was shown to reduce breast cancer stem cell characteristics and was associated with decreased expressions of CD44, CD133, ALDH1, Bod1, and BRCA2, and lower mammosphere formation while increasing sensitivity to radiation [31]. Another study showed that miR-142-3p represented a key therapeutic target for uveal melanoma [33]. Clinically, it was reported that the plasma level of miR-142-3p increased in patients with colon cancer after curative resection [34]. Our findings in this study corroborated the tumor-suppressive roles of miR-142-3p, in that transfection of CRC cells with miR-142-3p mimic molecules resulted in decreased expressions of LGR5, β-catenin, mTOR, and IL-6 as well as reduced SPs, as a surrogate of reduced ABCG2 expression. The reduction in the SP could function in concert with decreased expressions of LGR5 and β-catenin to re-sensitize 5-FU resistance in CRC cells.

Finally, using a CAF-educated DLD1 xenograft mouse model, we provide preclinical evidence for MSI-N1014 as a potential therapeutic agent for colon cancer. MSI-N1014-only treatment significantly delayed tumor growth compared to the vehicle and 5-FU-only groups. The combination of MSI-N1014 and 5-FU provided the highest suppressive effect on tumor growth, echoing the synergy demonstrated by our in vitro assays. The analysis of treatment-associated changes in body weights (BWs) of mice showed that there was no apparent difference in the median BWs of mice treated with MSI-N1014 alone or in combination with 5-FU during the entire duration of the experiment. The decreased LGR5, β-catenin, mTOR, and IL-6 levels, and increased miR-142-3p level in tumor samples collected from mice that received MSI-N1014, support our proposed anti-CRC mechanism of action. Finally, we showed that a higher level of miR-142-3p was present in pooled plasma samples from mice, which received MSI-N1014+5-FU and MSI-N1014-only treatments, compared to those of samples from the vehicle control and 5-FU-only groups. This observation is consistent with a previous report in which the miR-142 plasma level was higher in patients who received curative resection compared to their counterparts [34]. This provides support for the therapeutic/prognostic roles of miR-142-3p in colon cancer. Additional miRs are being investigated in our laboratory for their roles in colon tumorigenesis and response to post-MSI-N1014 treatment.

## 4. Materials and Methods

### 4.1. Cell Culture and Reagents

The DLD1 and HCT116 human colon cancer cell lines and normal fibroblasts (NFs, ATCC® PCS-201-018) were obtained from American Type Culture Collection (ATCC, Manassas, VA, USA) and were cultured according to the vendor’s recommended conditions. MSI-N1014 was synthesized as described previously in a U.S. patent application [12] (H.S. Huang, D.S. Yu, T.C. Chen, Vol. US Patent No. 8,927,717B1, US, 6 January 2015). Fluorouracil (5-FU) was purchased from SelleckChem (Hsinchu, Taiwan) (cat. no. S1209). Stock solutions of 5-FU and MSI-N1014 were dissolved in 10 mM dimethyl sulfoxide (DMSO; Sigma Aldrich, St. Louis, MO, USA) and kept at −20 °C. The stock solution was further immediately diluted in the sterile medium at the required concentrations.

### 4.2. Colon Tumor Sphere-Formation Assay

The tumor sphere-formation assay was performed according to a previously described method [35] with modifications. In short, colon cancer cells were seeded (2000 cells/well) in six-well ultra-low attachment plates (Corning, Corning, NY, USA) in serum-free media consisting of Dulbecco’s modified Eagle medium (DMEM)/Ham’s F12 (1:1), human epidermal growth factor (hEGF, 20 ng/mL), basic fibroblast growth factor (bFGF; 10 ng/mL (PeproTech, Rocky Hill, NJ, USA), 2 μg/mL 0.2% heparin (Sigma, St. Louis, MO, USA), and 1% penicillin/streptomycin (P/S, 100 U/mL, Hyclone, Logan, UT, USA). Cells were then allowed to aggregate and grow for at least 7 days. Cells (diameter > 50 µm), characterized by compact, non-adherent spheroid-like masses, were considered a tumor-sphere and counted with an inverted phase-contrast microscope.

### 4.3. Cell Viability Assay

The sulforhodamine B (SRB) assay established previously [36] was used to determine the efficacy of MSI-N1014 and 5-FU. Briefly, colon cancer cells (8000 cells/well) were seeded in 96-well plates and received different concentrations of MSI-N1014, 5-FU, or their combination for 48 h. Post-treatment, cells were fixed with 10% trichloroacetic acid (TCA) for 1 h at 4 °C. Plates were then washed with water and stained with 0.4% SRB (Sigma) in 1% acetic acid for 30 min at room temperature. Excess stain was removed by washing with 1% acetic acid twice. Plates were then air-dried overnight at room temperature, and the protein-bound stain was solubilized with a 20 mM Tris-base solution for 15 min on an orbital shaker. The absorbance was measured using a microplate reader at a wavelength of 515 nm.

### 4.4. Co-Culture and Enzyme-Linked Immunosorbent Assay (ELISA)

Human normal fibroblasts and colon cancer cells were co-cultured as follows. Colon cancer cells (HCT116 and DLD-1) were seeded into the upper compartment of a Transwell insert (Corning) at a density of 10^5^ cells/mL, with Normal fibroblasts (NF) also at 10^5^ cells/mL in the lower compartment. The experiments were divided into control and MSI-N1014 treatment (4 µm, DLD-1; 3 µm HCT116), and cells were cultured for 48 h. Subsequently, inserts containing cancer cells were removed. Fibroblasts in the lower compartment were then washed (phosphate-buffered saline (PBS), three times), and cultured in DMEM for an additional 24 h. The resultant fibroblasts (cancer-associated fibroblasts, CAFs) were then subjected to further analyses. The culture medium was then collected and measured using a VEGF ELISA Kit (R&D Systems, Minneapolis, MN, USA) and an IL-6 ELISA Kit (R&D Systems). The assays were performed according to the vendors’ protocols. For examining CAF-induced tumorigenic properties in colon cancer cells, CAFs generated as described above and parental DLD1 and HCT116 cells underwent similar co-culture conditions. After 48 h of co-culturing with CAFs, CAF-educated HCT116 and DLD1 cells were harvested for further analyses (including colony, tumor- sphere formation, migration, and drug resistance).

### 4.5. Immunofluorescence Imaging

In the immunofluorescence experiments, NFs and subsequently transformed CAFs were plated in six-well chamber slides (Nunc™, Thermo Fisher Scientific, Waltham, MA, USA) for 24 h. An immunofluorescence experiment was carried out using a previously established protocol according to vendor’s instructions. Primary antibodies were added and incubated at room temperature for 1 h. The primary antibody used was α-SMA (1:100, cat no. 48938; Cell Signaling Technologies). Matched secondary antibodies were anti-mouse immunoglobulin G (IgG) (H+L), F(ab’)_2_ fragment (1:800, AlexaFluor 488 conjugate, cat no. 4408; Cell Signaling Technologies, Taipei, Taiwan) and anti-rabbit IgG (1:600, AlexaFluor 555 conjugated, cat no. 4413). Stained cells were mounted using Vectashield mounting medium with 4,6-diamidino-2-phenylindole (DAPI) to counterstain the DNA. Cells were imaged with a Zeiss Axiophot (Carl Zeiss) fluorescence microscope. Microphotographs were captured using an AxioCam MRc digital video camera and analyzed using AxioVision Zeiss software (Carl Zeiss, Oberkochen, Germany).

### 4.6. Sodium Dodecyl Sulfate-Polyacrylamide Gel Electrophoresis (SDS-PAGE) and Western Blot Analysis

Total protein lysates from CRC cells (parental, tumor-spheres, and from co-culture experiments) were extracted after treatment in different experiments and were separated by SDS-PAGE using the Mini-Protean III system (Bio-Rad, Taiwan) and transferred onto polyvinylidene difluoride membranes using the Trans-Blot Turbo Transfer System (Bio-Rad). Membranes were incubated with the primary antibody to react overnight at 4 °C. Details of the primary antibody and dilutions used for these studies are listed in Table 1. Then membranes were incubated with the horseradish peroxidase-labeled secondary antibody. Proteins of interest were detected and visualized using enhanced chemiluminescence (ECL) detection kits (ECL Kits; Amersham Life Science, NJ, USA). Images were captured and analyzed using the UVP BioDoc-It system (Upland, CA, USA). The original western blots raw image can be found in Figures S1–S4.

### 4.7. Transient microRNA (miRNA) Transfection

The miScript miR-142-3p mimic, inhibitor, and negative mimic were purchased from (Qiagen, New Taipei City, Taiwan). DLD1 and HCT116 cells were seeded at 3 × 10^5^ cells/well in six-well plates and transfected using Invitrogen^®^ Lipofectamine^TM^ 2000 (cat. no. 11668019, Thermo Fisher Scientific., Carlsbad, CA, USA) according to the manufacturer’s protocol. After transfection, cells were harvested and further analyzed.

### 4.8. Drug Combination Index (CI) Evaluation

A quantitative measure of drug combination effects (MSI-N1014 and 5-FU), Chou-Talalay’s CI, was determined from experimental dose-response results [37]. Experimental data were processed and analyzed using CompuSyn, open-source software. Isobolograms for both DLD-1 and HCT-116 cells after treatment with different concentrations of 5-FU and MSI-N1014 were generated. CI < 1 indicates synergy; CI > 1 indicates antagonism, and CI = 1 indicates additivity.

### 4.9. RNA Isolation and Reverse-Transcription Polymerase Chain Reaction (RT-PCR)

Total RNA was isolated and purified using TRIzol-based protocol (Life Technologies) according to the protocol provided by the manufacturer. The RNA concentration and purity were determined with a NanoDrop 1000 spectrophotometer (Nyxor Biotech, Paris, France). One microgram of total RNA was reverse-transcribed using a Qiagen OneStep RT-PCR Kit (Qiagen), and the PCR was performed using a Rotor-Gene SYBR Green PCR Kit (400, Qiagen, Taipei, Taiwan). Details of qPCR primers used for this study are listed in Supplementary Table S1.

### 4.10. Wound-Healing Migration Assay

DLD1 and HCT 116 cells were resuspended in complete medium, plated in individual culture-inserts (ibdi, Munich, Germany), appropriated for a 2D migration assay, and maintained at 37 °C in a 5% CO_2_ atmosphere until confluence. These culture inserts were composed of two chambers separated by a biocompatible silicone material, which after removal allowed cells from each edge to migrate towards the center of the gap. After the barrier was removed, confluent cancer cell monolayers were washed with PBS to remove non-adherent cells, and treated with MSI-N1014. Treated and untreated cells were maintained at 37 °C in a 5% CO_2_ atmosphere for 24 h. Cell migration was evaluated every 2 h with the BioTek Lionheart FX automated cell imaging system to capture and monitor wound closure with a phase-contrast microscope [38].

### 4.11. Colony-Formation Assay

The colony-forming assay was performed according to a protocol described by Franken et al. [39]. Briefly, 300 colon cancer cells were seeded in six-well plates (Corning) and treated with MSI-N1014 (at the equivalent of the IC_10_ value 8–10 µM). After 7 days of incubation, the medium was removed, and cell colonies were fixed and stained with a crystal violet solution (0.1% crystal violet, 1% methanol, and 1% formaldehyde). Stained cells were washed with water and air-dried at room temperature. The number of colonies was quantified using a Cell3iMager neo scanner, and the percentage of drug-treated colonies relative to control colonies was calculated.

### 4.12. Flow Cytometry

Colon cancer cells (DLD1 and HCT116) were used to analyze the expression of CD44^+^. Briefly, 10,000 live cells were incubated with an anti-CD44 antibody (cat no. ab6124; dilution, 1:100; Abcam) at 37 °C for 30 min. After washing with PBS, cells were incubated with FITC-labeled goat anti-mouse IgG (cat no. F0257; dilution, 1:100; Sigma-Aldrich; Merck KGaA, Darmstadt, Germany) at 37 °C for 30 min. After washing three times with PBS, the fluorescence intensity was detected with a BD Accuri™ C6 Flow Cytometer with BD Accuri C6 software vers. 1.0.264.21 (BD Bioscience, San Diego, CA, USA). An SP analysis was performed according to a previously established method [40]. In short, SPs of HCT116 and DLD-1 cells (with different treatments) were determined using a FACSAria™ III sorter (BD Biosciences, Taipei, Taiwan). Verapamil (at a final concentration of 100 μM) and an ABCG2 blocker (which served as a control) were added to cell suspensions 15 min prior to incubation with Hoechst. SP cells, which expressed the ATP-binding cassette, ABCG2, and Hoechst 33342 efflux activity, were identified and determined to be SP cells.

### 4.13. In Vivo Evaluation of MSI-N1014

Immune-compromised NOD/SCID mice (6 weeks of age, females) were purchased from BioLASCO (Taipei, Taiwan). Animal experiments were conducted in strict compliance with the Institutional Animal Care and Use of Committee or Panel (IACUC/IACUP), Taipei Medical University (approval no.: LAC-2017-0161) protocol. First, the CAF-educated DLD1 cells and tumor-spheres (generated under serum-deprived conditions, 10^6^ cells/injection) were subcutaneously (s.c.) injected into the right flank of a mouse. Treatment was commenced when the tumor became palpable. Tumor-bearing mice were randomly divided into four different groups, 5 mice in each group: vehicle control, MSI-N1014-only (MSI-N1014, 10 mg/kg, five times/week), 5-FU only (5-FU, 10 mg/kg, three times a week), and the combination of MSI-N1014 (10 mg/kg, five times/week) and 5-FU (10 mg/kg three times/week); both agents were given intraperitoneally (i.p.). Change in tumor size (fold change) as indicated on the Y-axis = tumor size mm^3^ **_week X_**/ tumor size mm^3^ **_week 1_**), where x denotes the number of weeks post week one. Changes in body weight (BW) and survival were monitored on a weekly basis. After the experiment, mice were humanely sacrificed by cervical dislocation, and tumor samples were resected for further analyses.

### 4.14. Statistical Analysis

Three independent replicates were conducted in all experiments. Student’s *t*-test was used to evaluate the statistical significance. The Kaplan-Meier method was used for the survival analysis in the animal experiment. A *p*-value of < 0.05 was considered statistically significant.

## 5. Conclusions

In summary, we provide preclinical evidence to support the therapeutic functions of MSI-N1014. MSI-N1014 was shown to function in suppressing the colon cancer stemness markers, LGR5, and β-catenin, while also preventing the transformation of CAFs. In part, MSI-N1014-mediated antitumor effects acted through the induction of the miR-142-3p tumor suppressor. Further investigation is warranted for developing MSN-N1014 as a therapeutic agent.

## Figures and Tables

**Table 1:** List of antibodies.

Target	Dilution	Company and Catalog No.	Predicted MW (kDa)
GAPDH	1:1000	Proteintech, IL6 Rabbit mAb, 10494-1-AP	36
β-catenin	1:1000	Cell Signaling, β–Catenin (6B3) Rabbit mAb, #9582	92
mTOR	1:1000	Cell Signaling, mTOR (7C10) Rabbit mAb, #2983	289
LRG-5	1:1000	Epitomics, LRG5, Rabbit mAb, #2495-1	99.9
IL-6	1:500	Proteintech, IL6 Rabbit mAb, 21865-1-AP	24
EGFR	1:1000	Proteintech, EGFR Rabbit mAb, 18986-1-AP	165–145
ABCG2	1:2000	Proteintech, ABCG2 Rabbit mAb, 10051-1-AP	60–70
TGF-β1	1:1000	Proteintech, TGF-β1 Rabbit mAb, 18978-1-AP	25

## Supplementary Materials

The following are available online at https://www.mdpi.com/2072-6694/12/6/1590/s1, Figure S1: Raw western blot image for Figure 2E; Figure S2: Raw western blot image for Figure 3E; Figure S3: Raw western blot image for Figure 4E; Figure S4: Raw western blot image for Figure 5D; Table S1: Primer sequences of microRNA; and Supplementary Video S1: Wound healing assay.
