# Supplementary material for: Preclinical Evaluation of the Novel Small-Molecule MSI-N1014 for Treating Drug-Resistant Colon Cancer via the LGR5/β-catenin/miR-142-3p Network and Reducing Cancer-Associated Fibroblast Transformation
Source: Cancers (Basel). 2020 Jun 16;12(6):1590. doi: 10.3390/cancers12061590 (PMC7352915; doi:10.3390/cancers12061590)
Supplement: Supplementary file 1 [file cancers-12-01590-s001.zip › Supplementary Files/Supplementary Table 1-Primer sequences of microRNA.docx]

**Table 1: Primer sequences of microRNA**

| **miRNA** | **QIAGEN catalogue number** |
| --- | --- |
| hsa-miR-21-5p | MS00009079 |
| hsa-miR-22-5p | MS00009142 |
| hsa-miR-34a-3p | MS00009534 |
| hsa-miR-124-3p | MS00006622 |
| hsa-miR-26b-1 | MS00003234 |
| hsa-miR-142-3p | MS00031451 |
