# Supplementary material for: Preclinical Evaluation of the Novel Small-Molecule MSI-N1014 for Treating Drug-Resistant Colon Cancer via the LGR5/β-catenin/miR-142-3p Network and Reducing Cancer-Associated Fibroblast Transformation
Source: Cancers (Basel). 2020 Jun 16;12(6):1590. doi: 10.3390/cancers12061590 (PMC7352915; doi:10.3390/cancers12061590)

Western Blot Raw data

Figure S1 (2E) Raw WB data

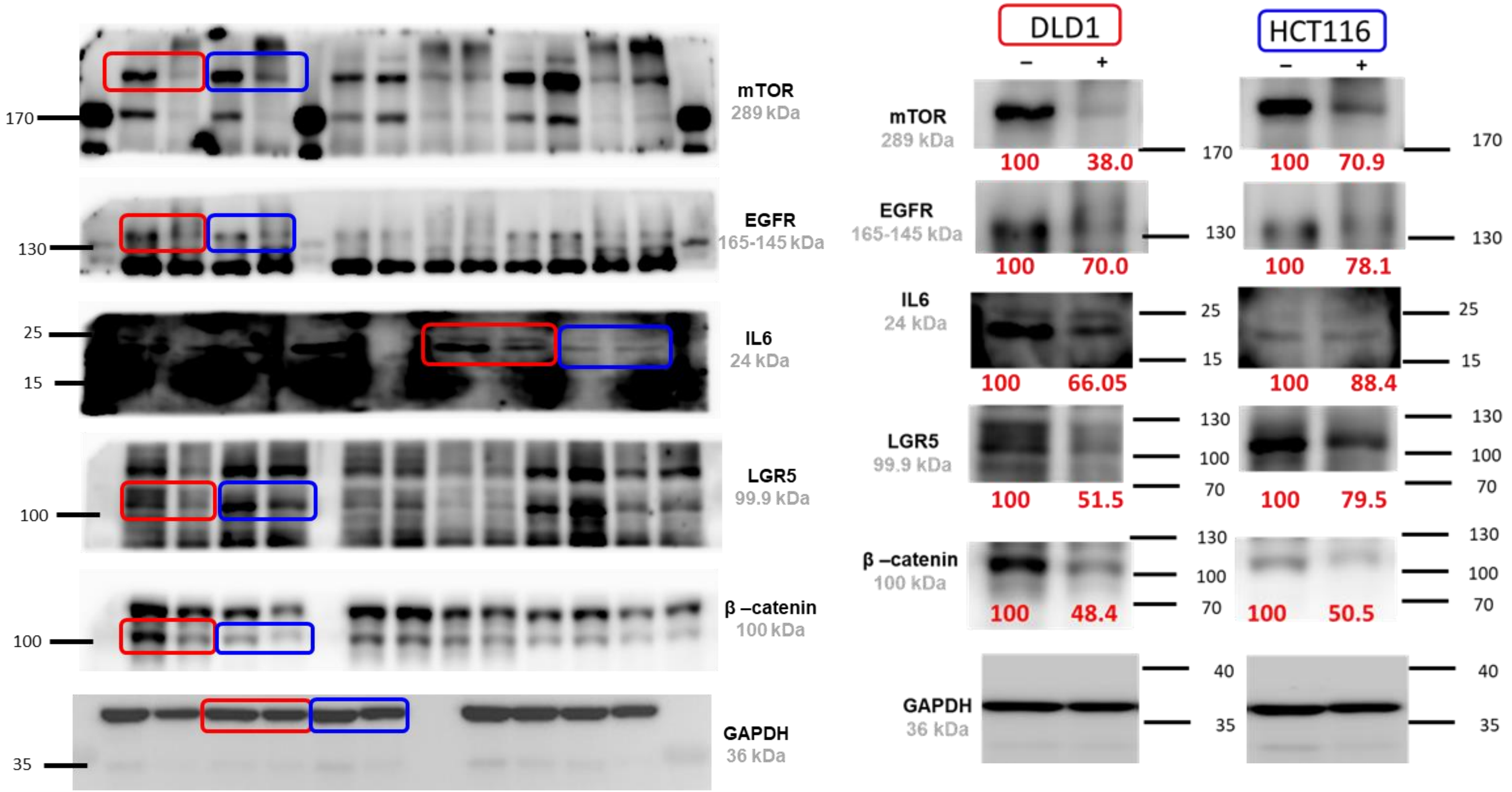

Figure S2 (3E) Raw WB data

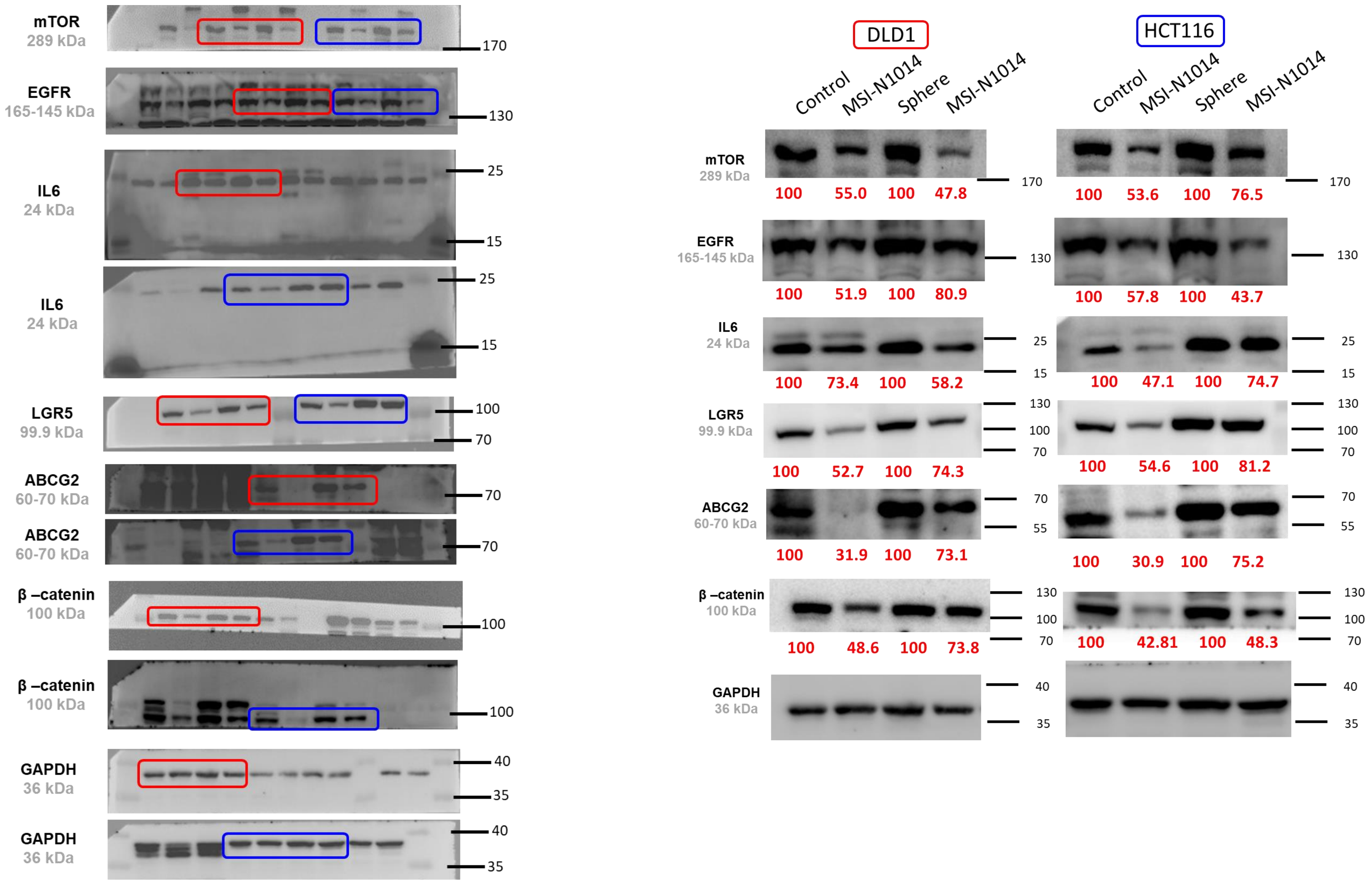

Figure S3 (4E) Raw WB data

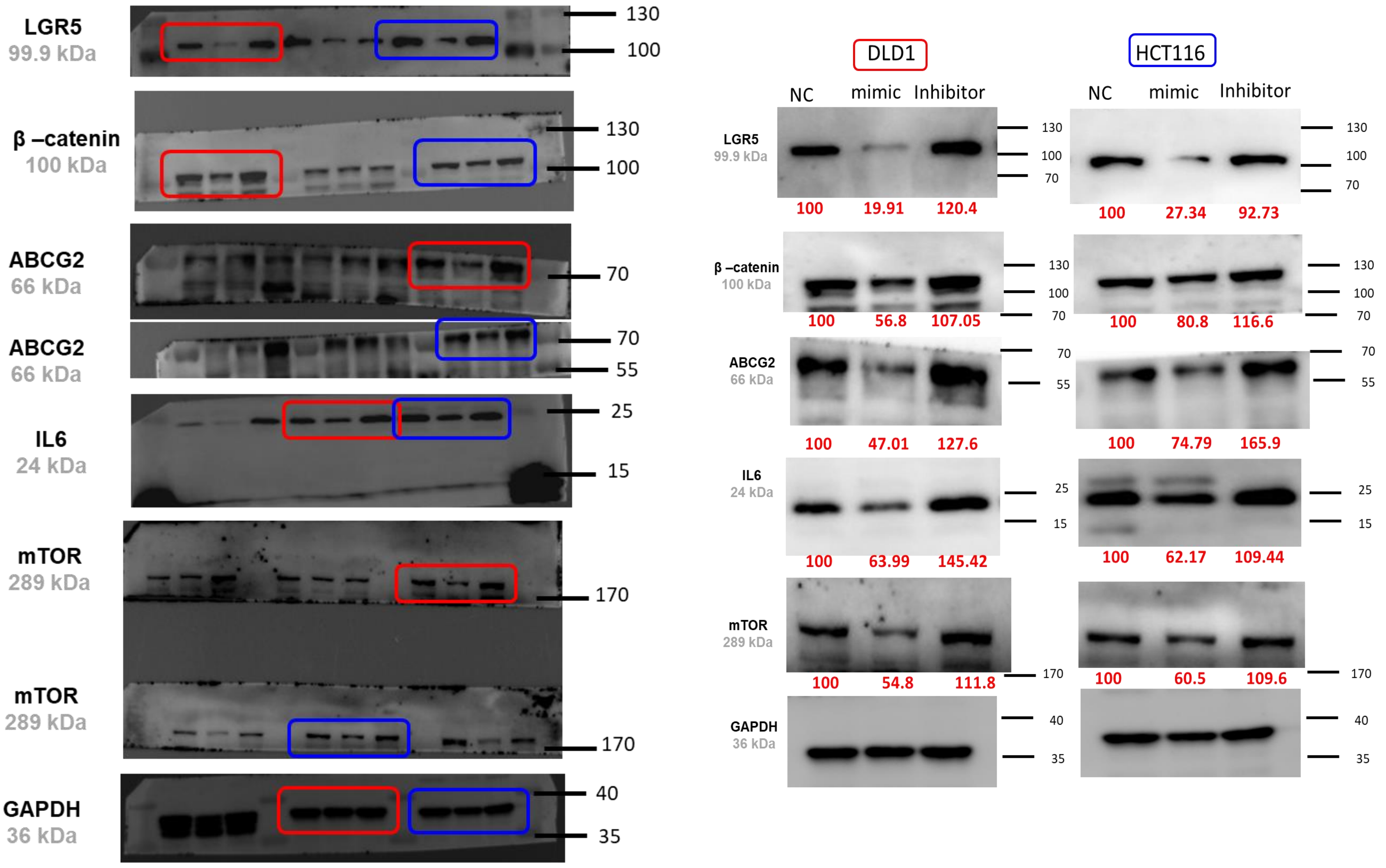

Figure S4 (5D) Raw WB data

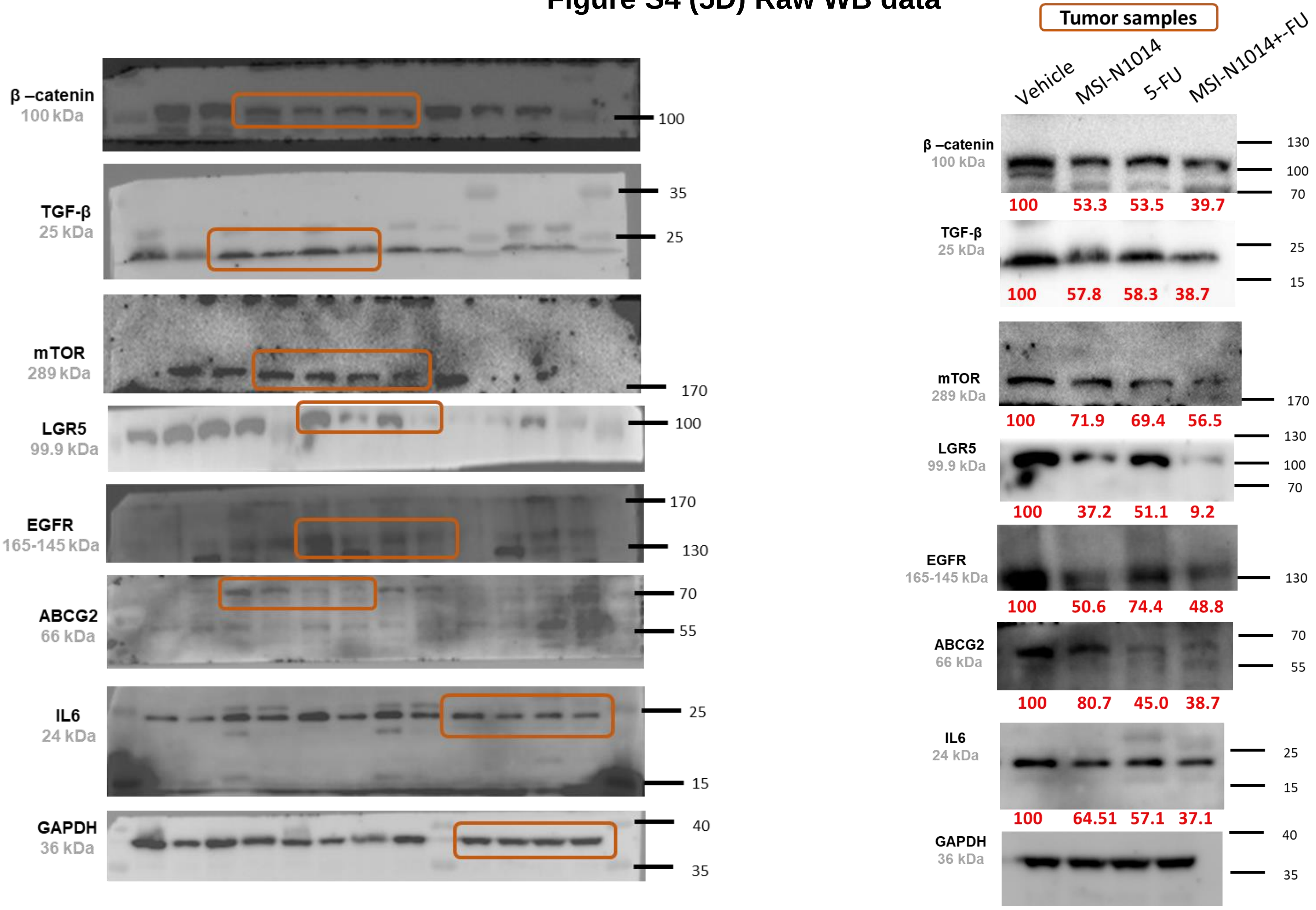

Supplement: Supplementary file 1 [file cancers-12-01590-s001.zip › Supplementary Files/20200616-MSI-N1014-Raw WB data_AW.pdf]
